# Laser-induced graphene gas sensors for environmental monitoring

**DOI:** 10.3389/fchem.2024.1448205

**Published:** 2024-10-31

**Authors:** Cadré Francis, Attila Rektor, Tony Valayil-Varghese, Nicholas McKibben, Isaac Estrada, Jennifer Forbey, David Estrada

**Affiliations:** ^1^ Micron School of Materials Science and Engineering, Boise State University, Boise, ID, United States; ^2^ Department of Electrical and Computer and Engineering, Boise State University, Boise, ID, United States; ^3^ Department of Biological Sciences, Boise State University, Boise, ID, United States; ^4^ Center for Advanced Energy Studies, Boise State University, Boise, ID, United States

**Keywords:** laser-induced graphene, volatile organic compounds, gas-sensing, environmental monitoring, sensor

## Abstract

*Artemesia tridentata* is a foundational plant taxon in western North America and an important medicinal plant threatened by climate change. Low-cost fabrication of sensors is critical for developing large-area sensor networks for understanding and monitoring a range of environmental conditions. However, the availability of materials and manufacturing processes is still in the early stages, limiting the capacity to develop cost-effective sensors at a large scale. In this study, we demonstrate the fabrication of low-cost flexible sensors using laser-induced graphene (LIG); a graphitic material synthesized using a 450-nm wavelength bench top laser patterned onto polyimide substrates. We demonstrate the effect of the intensity and focus of the incident beam on the morphology and electrical properties of the synthesized material. Raman analyses of the synthesized LIG show a defect-rich graphene with a crystallite size in the tens of nanometers. This shows that the high level of disorder within the LIG structure, along with the porous nature of the material provide a good surface for gas adsorption. The initial characterization of the material has shown an analyte response represented by a change in resistance of up to 5% in the presence of volatile organic compounds (VOCs) that are emitted and detected by *Artemisia* species. Bend testing up to 100 cycles provides evidence that these sensors will remain resilient when deployed across the landscapes to assess VOC signaling in plant communities. The versatile low-cost laser writing technique highlights the promise of low-cost and scalable fabrication of LIG sensors for gas sensor monitoring.

## 1 Introduction

*Artemisia tridentata* is a taxonomic group of native plants called sagebrush, which are largely found in the intermountain regions of western North America (Richardson et al., 2015). Sagebrush ecosystems play an integral role in the environment by influencing plant and animal species and reducing erosion (Prevéy et al., 2010; Duchardt et al., 2021; Williams et al., 2019; Zhu and Reed, 2012; Fusco et al., 2019). The distribution of sagebrush across the Great Basin region is larger than 75% of the countries worldwide and is threatened yearly by environmental variables such as wildfires, invasive grasses, and human disturbances (Coates et al., 2016; Ding et al., 2020; Requena-Mullor et al., 2023). There is significant interest in conserving the remaining intact sagebrush areas and restoring disturbed areas negatively impacted by human activities with sagebrush (Wang D. et al., 2022; Chambers et al., 2023; Maestas et al., 2022). However, targeted restoration management would benefit from the accurate classification of sagebrush for the selection of appropriate seed sources (Pickup et al., 2012). The distinct chemical profiles of sagebrush offer a potential phenotype to both classify species and populations of sagebrush and assess plant–environment and plant–animal interactions (Robb et al., 2022; Jaeger et al., 2016; Pezzola et al., 2017). Specifically, volatile organic compounds (VOCs) can classify sagebrush based on the environment, are critical for plant–plant communication, and influence interactions with herbivores of conservation concern (Jaeger et al., 2016; Karban et al., 2006; Ulappa, 2011; Frye et al., 2013). The capacity to continuously measure and quantify VOCs would provide a new understanding of how variations in the local conditions affect species interactions and provide a phenotypic biomarker for proactive conservation, restoration, and management efforts (Ricca and Coates, 2020; Melton et al., 2021). Here, we present an initial effort to advance the capacity to classify sagebrush, predict plant responses to environmental conditions, and predict species interactions by manufacturing sensors that can detect and quantify VOC emissions from sagebrush.

Graphene is synthesized via several methods, including chemical vapor deposition, chemical exfoliation, mechanical exfoliation, laser-induced graphene (LIG), and epitaxial growth (Li et al., 2009; Park and Ruoff, 2009; Yazdi et al., 2016). Among them, LIG is an economical choice in terms of energy and cost when compared to traditional fabrication methods. LIG is a 3D porous graphitic material with a high surface area and good electrical conductivity, proving suitable for applications in gas sensing. LIG, formed by laser irradiation of a carbon-rich material, induces a photochemical and thermal conversion into graphene. This one-step approach uses a CO_2_ infrared laser to photothermally convert sp^3^ hybridized carbon atoms within the polyimide film to sp^2^ due to the change in localized temperature, hence producing a material with high electrical conductivity (Lin et al., 2014; Wang H. et al., 2022). This process offers a cost-effective and scalable synthesis route for graphene synthesis, enabling the creation of a 3D porous graphitic material made by direct writing on a carbon-rich precursor with a laser (Lin et al., 2014; Vivaldi et al., 2021).Thermal, mechanical, and electrical properties of the material have been an area of interest for both experimental and theoretical studies (Novoselov et al., 2004; Novoselov et al., 2007; Bharech and Kumar, 2015). Laser synthesis allows ease of design and surface characteristics as CAD models and laser power are easily changed, allowing application-specific models to pattern flexible substrates. The direct-write laser synthesis allows for the material to be patterned in desired geometries and have a high sensitivity due to the porous nature created by the evolution of gases during the process (Wang H. et al., 2022). The material fabrication technique allows the use of carbon-rich precursors to form high-quality graphene micropatterns; this can be changed by ambient conditions during the synthesis, which add functional groups to the surface to modify the performance (Vivaldi et al., 2021; Wang et al., 2018). As no specific raw materials are needed, any carbon-rich substrate is suitable to act as an LIG substrate. This versatility allows the process to be green by using renewable precursors such as food, cloth, and paper (Chyan et al., 2018). The graphitic structure created is dependent on the molecular structure of the starting material, which affects the properties of the resulting LIG. Simulations studying the use of common polymers to create LIG find an initial amorphous structure growing into an ordered graphitic structure with altered carbon rings that may be affected by the number of cycles (Vashisth et al., 2020; Chen et al., 2019). Modeling work has been conducted to determine the effect of laser irradiation on polyimide. The heating process was mimicked, showing that increasing temperature during irradiation leads to a pressure of approximately 3 GPa at annealing temperatures ranging from 2400 K to 3000 K, forming the crystal structure (Dong et al., 2016).

The defect-rich nature of LIG, caused by the rapid evolution of gases, can lead to a modified lattice including five- or seven-membered carbon rings distorting the lattice (Lin et al., 2014; Vivaldi et al., 2021). These defects allow the capture of electrons within the carbon rings along with an ordered porous morphology, enhancing the surface area available within the material (Lin et al., 2014; Wang et al., 2018). Surface area studies of flat LIG using the Brunauer–Emmett–Teller (BET) method have found flat LIG’s surface area to be ≈ 340 $\frac{m^{2}}{g}$ with pore sizes of less than 9 nm and fibrous LIG to have a surface area of <70 $\frac{m^{2}}{g}$ (Lin et al., 2014; Vivaldi et al., 2021; Duy et al., 2018). Line density, laser power, and laser focus (beam diameter) are all important factors in controlling the surface area of LIG. A recent study showed that the surface area decreased from ≈370 $\frac{m^{2}}{g}$ to ≈100 $\frac{m^{2}}{g}$ with 8.2 W used instead of 1.7 W using a CO_2_ laser; the total area of LIG pores in the film obtained also varied from ≈365 to 660 (Zonov et al., 2024). This high surface area due to the abundance of defect sites and structure of the bonds allows control of the electron transfer chemistry within the material (Sharma et al., 2010). Defects have been functionalized in graphene derivatives such as graphene oxide to improve the catalytic activity; the carboxylic groups at the edges of defects with localized unpaired electrons allowed for sites to trap molecules (Su et al., 2012). This structure and the density of defects have been found to improve the selectivity of the material, with the high defect concentration providing an abundance of active sites, while maintaining good electrical conductivity (Cheng et al., 2022). VOC detection with nanomaterial sensors has shown promise in gas detection with a focus on controlling the morphology produced in the synthesis and functional modification leading to the targeted electronic properties (Wang et al., 2020).

Previous studies have shown the suitability of graphene for gas sensing applications due to the high surface area-to-volume ratio and the effect of chemisorption and physisorption on the electronic properties (Stanford et al., 2019). The high surface area (350 m^2^g^-1^) and porous structure supply many sites for gas–solid interactions (Lin et al., 2014). Gas detection capabilities have been demonstrated using NO_2_, as an active electrode in an MoS_2_ gas sensor. The morphology of the MoS_2_ increased the sensitivity of the LIG to detect the presence of the gas acting as a chemiresistive sensor relying on direct charge transfer (Yan et al., 2020; Yi and Cheng, 2020). The chemical sensitivity of LIG can also be increased by functionalizing the surface with materials that selectively bind to the analyte of interest. Nitrogen has been measured by functionalizing LIG electrodes using NH_4_^+^ and NO_3_^−^ ion-selective membranes to quantify the concentration in soil (Garland et al., 2018) and utilizing zinc nanorods with large specific areas to provide a sensing layer for NO_2_ gas (Tseng et al., 2023). LIG sensitivity to gases has been measured by measuring the resistance when exposed to air after being held in vacuum (Stanford et al., 2019), self-heating LIG with dispersed nanomaterials with different selectivity (Yi and Cheng, 2020), and increasing the analyte concentration over time (Yang et al., 2022). Due to the complexity of volatile organic compounds in sagebrush, there are very few solutions to measure real-time emissions of VOCs in sagebrush ecosystems. A calibrated electronic nose or the use of headspace chromatography are the methods commonly used to differentiate among VOCs and classify species of sagebrush and their responses to environmental changes (Jaeger et al., 2016; Laothawornkitkul et al., 2008). Electronic noses are complex systems that contain an array of sensors and use algorithms for aroma classification. Although electronic noses are cheaper than chromatography or mass spectrometry techniques, they may not be able to identify individual chemical species (Wilson, 2018). These systems also require pattern recognition systems and other techniques to improve the selectivity, sensitivity, robustness, and reversibility (Saeed et al., 2009).

These methods cannot offer continuous monitoring and only provide a snapshot of the chemistry when the measurements are taken or the samples are collected. LIG sensors could be used to provide real-time continuous evaluation of sagebrush chemistry due to their high-surface area fast response time to gases (Stanford et al., 2019). Wireless communication has been used with a laser-induced graphene paper hybrid structure, showing that the energy requirements for LIG-based sensors are low and variance in resistance can be communicated reliably (Jung et al., 2022). That sensor was used to continuously monitor the status of food and temperature of water, and the ability to use carbon-rich precursors to directly pattern these devices was beneficial to that application. The initial work was conducted investigating using Bluetooth to monitor LIG VOC sensors (Mark et al., 2023). These sensors can be generated rapidly and for low cost, making them viable for VOC detection across a large area of interest. They are lightweight and can be mounted on plants, directly communicating readings at different times of the day. LIG sensors offer a new opportunity to investigate chemical phenotypes resulting from genome-by-environment interactions (Ninkovic et al., 2019). These sensors could enable the monitoring of sagebrush emissions which are used to understand species as well as the health of these plants, which also help understand the health of the total ecosystems and the animals that depend on it. This sensor is used to measure chemical signals released by these plants as a result of environmental conditions and play an important role in understanding the environmental adaptation of plants within a changing social–ecological region (Ninkovic et al., 2021).

## 2 Materials and methods

### 2.1 Materials

Polyimide (Kapton; 1.8 mil) film was purchased from DuPont, silicon dioxide wafers were acquired from University Wafer, and the polyimide solution was prepared via a polycondensation reaction from polyimide flakes. The 450-nm 7 W laser was purchased from Amazon (MYSWEETY 2 in 1 7000 mW CNC 3018 Pro Engraver Machine), and the Laurell WS-650 spin coater is used to spin-coat on silicon coupons. A Keithley 4200-A SCS parameter analyzer is used to conduct the electronic characterization of the material and sensor. A custom-built bend testing apparatus was used to test the effects of mechanical stimulations on the electrical property reliability in the LIG material. FEI Teneo SEM and HORIBA Raman spectroscopy were used to perform the structural characterization of the synthesized material. AutoCAD software was used to design the LIG electrode pattern.

### 2.2 Substrate preparation step 1

The silicon wafer was diced into 1 cm × 1 cm coupons. These coupons were bath-sonicated in acetone at 50°C for 10 min. The coupons are then removed from acetone and sequentially sonicated first in isopropanol and then in deionized water for 10 minutes. This material was spin-coated at 500 rpm for 30 s and then 1,500 rpm for 1 min; four layers of polyimide were spin-coated on each coupon.

### 2.3 Substrate preparation step 2

Polyimide film (Kapton) of 1.8 mil thickness was cleaned using a lint-free cloth and isopropanol. The Kapton is then mounted and taped on all sides to the laser engraving plate.

### 2.4 Common steps

Laser irradiation using the 450-nm visible light laser transforms polyimide-based substrates into laser-induced graphene. Polyimide shows much absorption near the UV region, allowing the carbonization of carbon-rich materials at various laser powers (Garland et al., 2018; Li, 2020). The pattern was created using AutoCAD software to prepare the design so that patterns were created with electrical, surface, and material properties for our intended use. The CAD designs were then exported to a LaserGRBL-compatible format, which was used at varying laser powers and focus distance on both the polyimide tape and the polyimide film that was spin-coated on the silicon substrate. The AJP was then used to print nanoparticle-based silver ink to function as electrodes on the LIG sensors.

### 2.5 LIG characterization

An array of van der Pauw structures was created by changing the line density (lines/mm) and laser power (W) of the laser; an array with the same parameters was also used to create samples for Raman and SEM analyses. These settings were selected after doing initial work characterizing the properties and performance of synthesized LIG. 1 $\frac{line}{mm}$ , 5 $\frac{lines}{mm}$ (0.2), 10 $\frac{lines}{mm}$ (0.1), 15 $\frac{lines}{mm}$ (.067), and 20 $\frac{lines}{mm}$ (0.05) were chosen; these line densities will be listed in the order of increasing line density from A–E. Power settings of 10% (0.7 W), 15% (1.05 W), 20% (1.40 W), 25% (1.75 W), and 30% (2.10 W) were used and are listed in the order of increasing power from 1 to 5, as reflected in Table 1.

**TABLE 1:** Corresponding values assigned to line density and power lasing parameters. The letters A–E denote values related to line density with E being the highest density; the numbers 1–5 designate the wattage of the laser used, with 5 being the highest laser power.

Sample	Line density (line/mm)	Laser power (W)	Power (number equivalent)
A	1	0.70	1
B	5	1.05	2
C	10	1.40	3
D	15	1.75	4
E	20	2.10	5

### 2.6 Raman spectroscopy

A 532-nm laser was used to characterize the LIG material synthesized at various laser powers. The ID/IG peak ratios at the different laser powers for both substrates were used to correlate laser power with the crystallite size and layer numbers (Cançado et al., 2006; Pandhi et al., 2020).

### 2.7 Scanning electron microscopy

FEI Teneo was used to acquire high-resolution images of each sample’s morphology. Planar views of the samples were collected using an accelerating voltage of 10 kV and a secondary electron detector, while cross-sections were obtained using a 2 kV voltage and an Everhart–Thornley detector. Magnifications were chosen so that at least one full laser raster was included in each image, with the horizontal field width (HFW) held constant. To avoid charging effects, an electrical pathway was added through a conductive adhesive copper tape.

### 2.8 Van der Pauw

Four-point probe measurements were conducted using the van der Pauw cloverleaf geometry samples of LIG using Keithley 4200. As LIG is created using a laser irradiation process, the properties of the material are sensitive to the surface. To conduct this study, four contacts were placed at the four edges of the structures. Current flowed between the top two contacts, and the voltage drop between the adjacent pair was measured. This previously described step was repeated, flowing current between contacts 1–3 and measuring the voltage drop between contacts 2–4. This provided both vertical and horizontal resistance information for the LIG structures.

### 2.9 Bend testing

Bend tests were conducted at room temperature, and the sensor with the best electrical performance was tested. The samples were repeatedly bent at radii of 10 mm and 15 mm to measure electrical resistance. The characteristics of the LIG material were studied using serpentine patterns and collecting resistance measurements every 10 bends up to 100 bending cycles.

### 2.10 VOC response testing

The Keithley 4200 probe station was used to measure the resistance changes in the presence of VOCs. Sagebrush leaves were heated to 30°C to promote the release of these VOCs, and resistance was measured by connecting leads from the sensors to the probe station. The container holding the leaves is then covered with parafilm to allow any VOCs to desorb from the LIG material. Resistance changes are then used to quantify the concentration being measured by the LIG sensor.

## 3 Results and discussion

**FIGURE 1:**
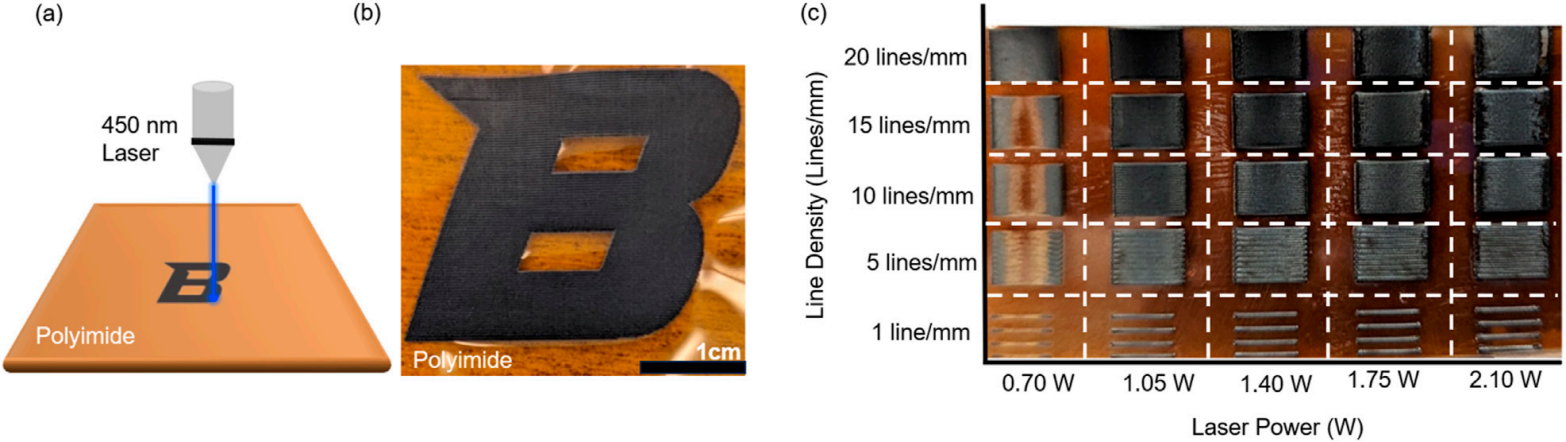
**(A)** Schematic of the fabrication of LIG from polyimide. **(B)** Optical image of LIG patterned into a Boise State “B” shape. **(C)** Optical image of the LIG array patterned in a rectangle shape. Each structure in the array corresponds to a specific laser power and line density. The data provide insights on threshold processing parameters to create an LIG structure.

Figure 1A shows the irradiation of the polyimide film to create a pattern using a 450-nm laser. This process creates the patterned LIG structure depicted in Figures 1A, B, and due to a laser being used for fabrication, complex LIG patterns can be created with designs completed in CAD, JPEG, or GCODE formats. To understand the formation of LIG on polyimide films, the 450-nm laser source with varying line density and power was applied on the substrate, as shown in Figure 1C.

**FIGURE 2:**
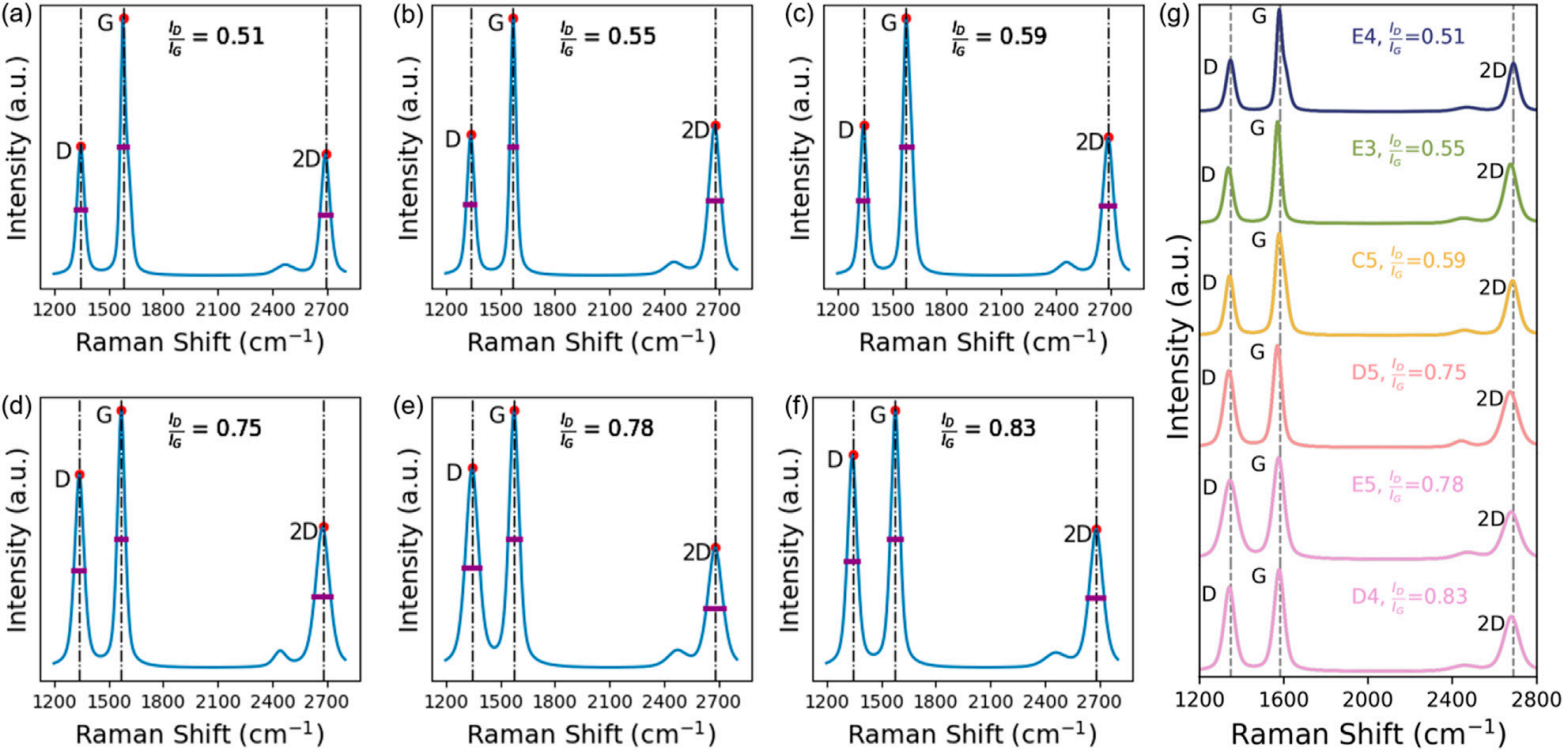
**(A–F)** Raman spectra obtained for six samples with the best electrical properties. The representative **(D, G)** and 2D peaks are labeled, red circles are used to denote the relative positions of each peak, and a purple bar is used to denote the calculated full width at half maximum (FWHM) of the characteristic graphene peaks. The ID/IG ratio is used to characterize the defect density of the LIG samples. **(G)** A stacked Raman plot is used to show the peak positions of six samples with the best electrical properties. Vertical lines are placed at expected peaks to show slight Raman shifts within the samples, and the vertical lines are placed at the expected D peak:1,350 cm^-1^, G peak: 1,583 cm^-1^, and 2D peak: 2690 cm^-1^.

The LIG gas sensor was designed by fabricating a serpentine and polygon sensing region with silver paste mounted to provide a secure connection for electrical characterization. The laser irradiation with a 450-nm laser produced continuous multilayer LIG structures characterized. Samples E4, E3, C5, D5, E5, and D4 are plotted in Figures 2A–F to show the effect of fabrication settings on the defect density (ID/IG) of the synthesized material. These samples were chosen as the best results of the array; other samples showed characteristics with more graphitic characters, while some others caused ablation of polyimide at the edges of the structure, as shown in Figure 1C. This ablation is likely the result of heat not dissipating fast enough as the raster direction changes.

Raman spectroscopy is a powerful technique for this analysis due to its well-studied ability to identify the structure including the D-band (disordered structure of graphene), G-band (C–C bond present in graphitic materials), and 2D-band (resonance structure of graphene produced by sp2 bonding present in carbon materials) (Childres et al., 2013). The Raman spectra for the samples were characterized and are plotted in Figure 2G; this plot shows the slight shift in peaks due to the processing parameters. All samples exhibited the D (≈1,350 cm^-1^), G (≈1,583 cm^-1^), and 2D (≈2690 cm^-1^) peaks, confirming the presence of LIG. The D-band shows the disordered structure of the LIG material and is due to defected phonons, the G-band shows the first-order phonons, and the 2D band is due to second-order phonons linked to the electronic band structure (Ferrari et al., 2006). The characteristic Raman peaks are present; it shows that the material is nanocrystalline rather than amorphous in nature (Childres et al., 2013; Ferrari et al., 2006).

**FIGURE 3:**
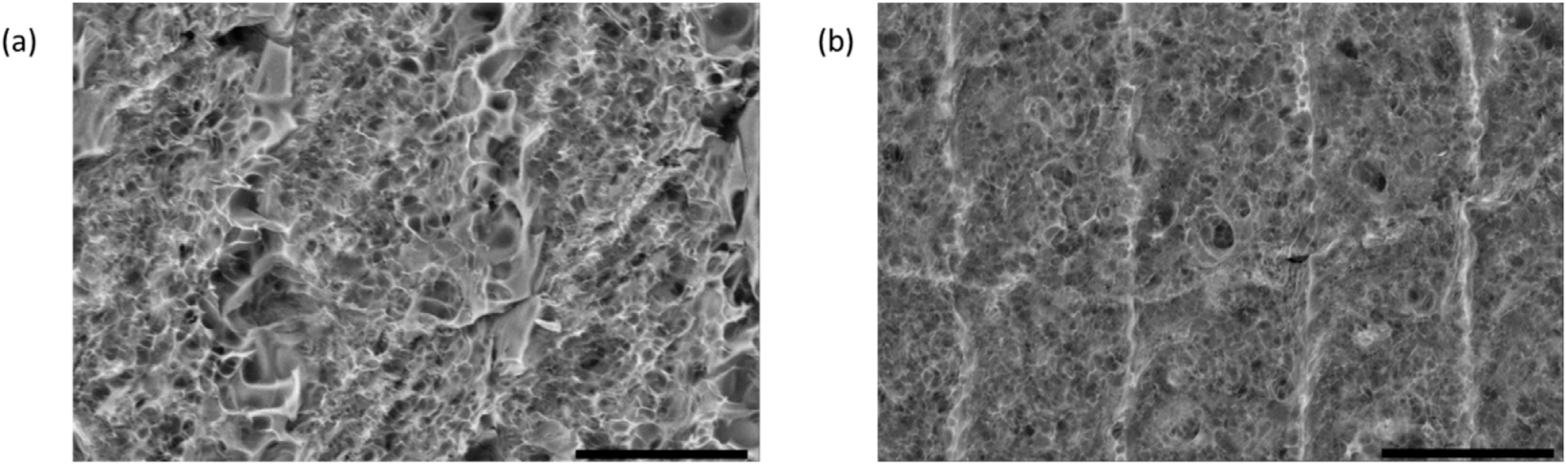
SEM images of LIG on the polyimide film; scale bars are 50 microns in length. **(A)** LIG sample at 200 micron horizontal field width (HFW) showing a porous structure created at 2.10 W and 15 line/mm (D5). **(B)** LIG sample at 200 micron horizontal field width (HFW) showing a porous structure created at 1.40 W and 20 line/mm (E3).

SEM images obtained from samples D5 and E3 were obtained via FESEM, as shown in Figures 3A, B. These images reveal that the surface of the synthesized material is a sponge-like structure, porous in nature exhibiting a high surface area. This morphology is due to the rapid evolution of gases caused by rapid irradiation of the polyimide precursor (Vivaldi et al., 2021). This surface area provides areas for gas–solid interaction and is beneficial for being able to measure the sensor response. At low powers and low line density, the gap between lasers is evident, and these areas show that LIG does not form a uniformed, continuous structure. At medium power and line density, a highly defective area is visible between the raster patterns. These samples also show more porosity, and the direction of the raster can be seen as well as the higher level of uniformity between the rastered lines. High power and high line density show the most porosity within the LIG structure. The images show full conversion of the polyimide precursor with a highly defective and porous nature. The uniform films are more conductive due to the complete graphitization of the amorphous precursor to crystalline LIG.

**FIGURE 4:**
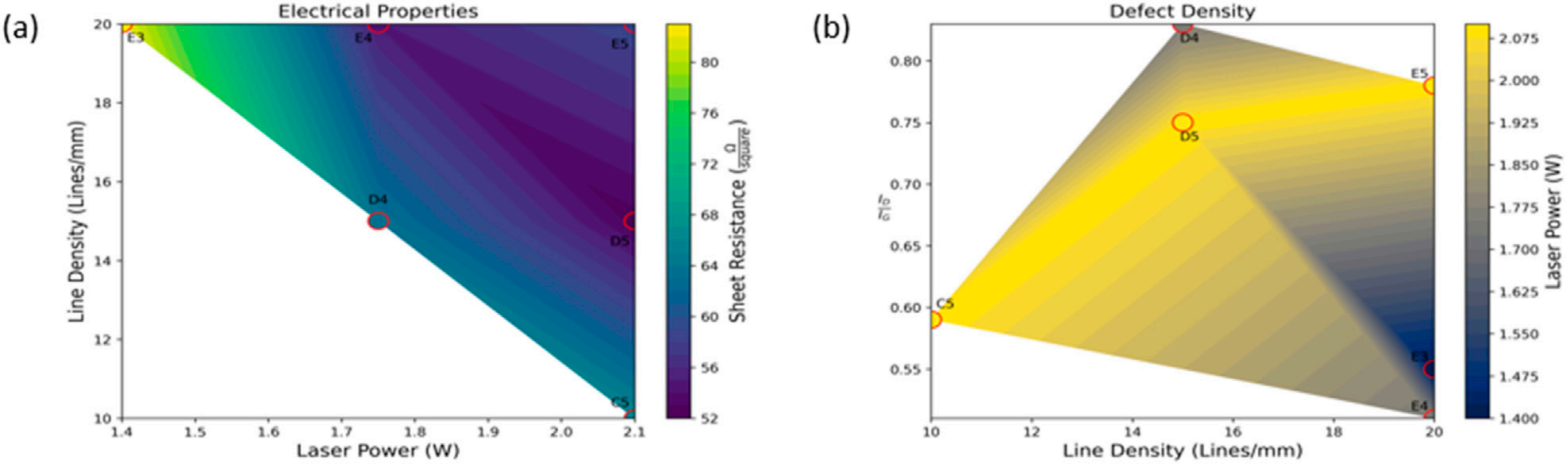
Electrical properties and defect density as it relates to LIG fabrication parameters shown in Table 1 and Figure 1C. **(A)** Sheet resistance of LIG samples related to laser parameters. **(B)** Defect density of LIG related to laser parameters.

Electrical resistance is an important characterization technique for 2D materials. In this work, I–V measurements are used to measure the sheet resistance of the fabricated LIG material. Higher resistance requires a larger change in the presence of sagebrush VOCs to quantify the differences. The ability of these sensors to detect the presence of VOCs is beneficial for applications in environmental monitoring. The aim was to create a complex LIG pattern capable of a low sheet resistance with a high surface area to create a functional device. The silver nanoparticle ink printed on both serpentine patterns and van der Pauw structures allow for electrical characterization with minimal contact resistance. This allows the probes to be directly mounted on the silver material to enable current supply and voltage measurements across all electrodes. Horizontal and vertical resistance measurements are taken, followed by the sheet resistance being calculated using the van der Pauw equation $\left(e\frac{-\pi R_{vertical}}{R_{s}}+e\frac{-\pi R_{vertical}}{R_{s}}=1\right.$ ). Sheet resistance measurements for samples E4, E3, C5, D5, E5, and D4 ranging from 52–82 $\frac{Ω}{square}$ are shown in Figure 4A. These measurements were repeatable, and small differences in sheet resistance are likely a result of the laser’s direction of travel. Laser line density and laser power both affect the sheet resistance; a large increase in sheet resistance was found in both 5 and 10 line/mm fabrications. The highest sheet resistance was found in the 2.1 W sample at a 5 line/mm density. The sheet resistance of this sample was 1.4 kΩ/square due to a non-continuous film. The laser power of 0.7 W with 20 line/mm density was measured at 409 $\frac{Ω}{square}$ , which is approximately five times higher sheet resistance than the same line density at 1.4 W. This shows the incomplete conversion of the polyimide starting material to LIG. ID/IG peak intensity ratios are used to characterize the level of disorder within the graphene structure. In the samples tested, this ratio was over a range of 0.51–0.83, showing a high defect density that can be seen related to processing parameters shown in Figure 4B.

**FIGURE 5:**
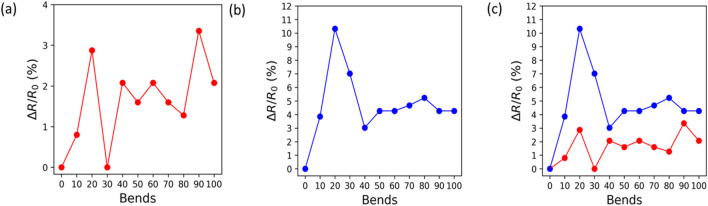
**(A)** Change in electrical resistance vs. number of bend cycles with a 15 mm bend radius of the E3 sample for 100 bend cycles. **(B)** Change in electrical resistance vs. number of bend cycles with a 10 mm bend radius of the E3 sample for 100 bend cycles. **(C)** Resistance increases due to the number of bend cycles for both E3 samples; 15 mm bend radius and 10 mm bend radius are shown in red and blue, respectively.

Figures 5A–C show variations in electrical resistance as the function of the number of bending cycles. Mechanical testing of these LIG films is useful as they will be deployed in real-world applications rather than in a laboratory environment. To ensure that the sensors are not susceptible to electrical degradation, they are evaluated as a function of the bending radius and number of bending cycles. The resistance $\left(\frac{\Delta R\;}{R_{0}}\right)$ of tested films remains within 10% of the normalized resistance up to 100 cycles with a bending radius of 15 mm. Raman characterization was also conducted post analysis to see if bending affected the Raman spectra. As crystallinity increases, the resistance to bending degrades (Wang et al., 2021). There was no shift in the Raman spectra obtained after mechanical testing, showing the integrity of the LIG material. A study was also conducted in which the raster direction was parallel to the bending radius rather than perpendicular; these results show similar electrical performance. The loading direction does not have a quantifiable effect on the microstructure changes due to bend cycles.

**FIGURE 6:**
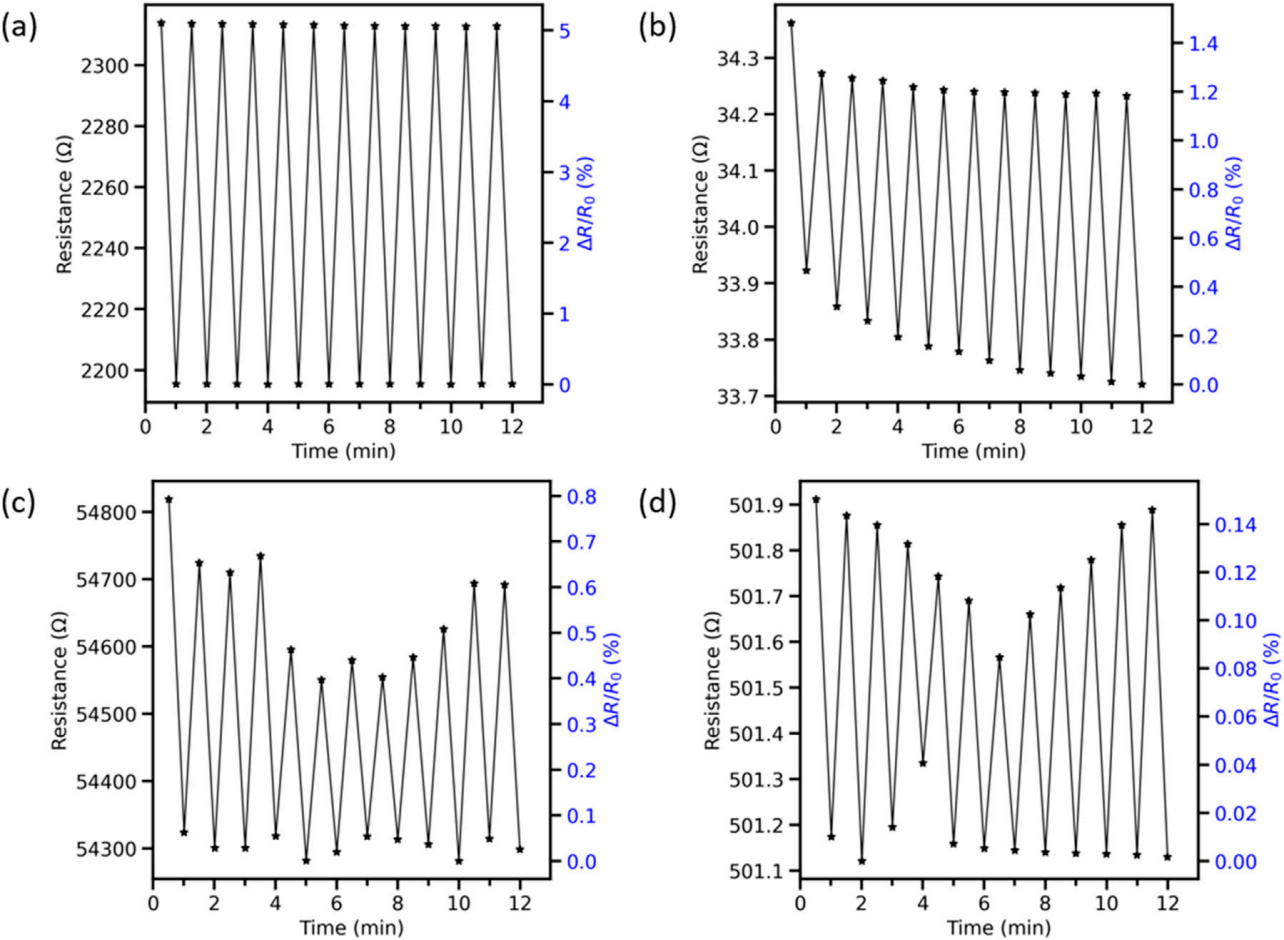
Dual-axis plots showing the response of LIG gas sensors to sagebrush leaves over a period of 12 min for the following sensors: **(A)** LIG patterned on Kapton in a serpentine pattern. **(B)** LIG patterned on Kapton in a polygonal shape. **(C)** LIG serpentine shape patterned on spin-coated polyimide on SiO_2_. **(D)** LIG polygon shape patterned on spin-coated polyimide on SiO_2_.

The resistance of LIG during exposure to sagebrush leaves was measured to quantify the chemiresistive gas sensing properties of the material. Silver was chosen for sensor connections due to the low electrical resistance and resilience to degradation under normal conditions. An initial resistance of the LIG sensors was taken to establish a baseline, and this allowed the analysis of repeatability after exposing the sensors to sagebrush and starting a 30-s measurement cycle. LIG sensors were fabricated with both serpentine and polygon patterns to measure how the shape of the active material may affect the device performance. The serpentine-patterned LIG on Kapton had the best performance in comparison with that of the other sensors, showing a fast response and a ≈5% and consistent recovery when cycled over 12 min. In comparison, the spin-coated polyimide had an average response of 0.6%. The four LIG-based sensors showed a fast response; however, the other three had lower resistance change and repeatability varied, as shown in Figures 6A–D. The LIG patterned on Kapton had a higher response due to the surface area and thickness of the material, offering more surface area for gas–solid interactions. The polygon-patterned LIG sensor showed a response of more than 1% in comparison with the minimal response of the spin-coated polyimide sensor with the same geometry, showing a very low response of ≈0.1%. LIG sensors produced on both types of polyimide precursors show a high repeatability, making the material usable and practical for sagebrush VOC sensing. Increasing the sensitivity of these sensors would be an important factor, and it may be improved by tuning the geometry of the LIG sensor. LIG gas sensors have shown rapid response to gas molecules due to the high surface area and a high sensitivity and selectivity when exposed to various gases such as air, helium (He), and oxygen (O_2_). The sensor response to helium gas showed repeatability for over 50 cycles, demonstrating efficiency in differentiating gas concentration changes within gas mixtures (Stanford et al., 2019).

Moisture poses a challenge for LIG-based gas sensors. A semi-permeable polydimethylsiloxane (PDMS) membrane has been used to repel water and other aqueous components in the air. In a study measuring NO_x_ absorption, the use of this material hindered the degradation of the sensitivity in the presence of moisture, although at the cost of decreased response/recovery times (Yang et al., 2022). Although there are minor variations in each measurement, there is no degradation in the signal response of the sensor showing robustness in operation; more complex patterns of LIG manufactured on Kapton could increase the sensitivity of the sensor and provide higher resolution related to the analyte being measured. The sensor operated at higher than ambient conditions due to sagebrush leaves being heated; the increased temperature improved the desorption of gas molecules from the active material, allowing the resistance to return to close to baseline values when cycled.

Further testing of these sensors and quantifying the response and recoverability of the sensor in a controlled environment with exposure to different species of sagebrush will improve the accuracy of the sensor in order to measure the response to environmental stressors. These sensors provide a lightweight, wearable solution to measure VOC behavior in real-time to further understand the dynamic chemical signals exhibited by the composition and concentration of VOCs resulting from genomic interactions in the sagebrush steppe.

## 4 Conclusion

This paper demonstrates a one-step additive manufacturing approach for the fabrication of low-cost and scalable LIG sensors for use in environmental monitoring. The LIG gas sensors fabricated using a 450-nm laser were used to effectively measure the presence of VOCs expressed from sagebrush. Use of a low-cost precursor showed a low resistance of approximately 2 kΩ with high reversibility and repeatability to measure the presence of sagebrush. Experiments showed fast response times and increased resistance due to the adsorption of VOCs to the LIG sensing material. The high porosity of the LIG material and the morphology of the structure provide many active sites for gas–solid interactions. Sensitivity can be improved by fabricating gas sensors with more surface areas within the same small, easily deployable package. Further work quantifying the sensor response to specific compositions of LIG would provide additional information on sagebrush species being measured. The fabrication parameters of LIG have a direct influence on the electrical properties of the material and its suitability for the VOC testing application. This is a low-power sensor and may be integrated using Bluetooth to monitor remote sagebrush ecosystems.

The real-time monitoring of the sagebrush response to the environment allows a greater understanding of the success of plants within this habitat. This will help in the restoration of a large area of the sagebrush ecosystem destroyed by fire and other activities.

## Data Availability

The original contributions presented in the study are included in the article/Supplementary Material; further inquiries can be directed to the corresponding author.
